# Simulation of nitrate pollution and vulnerability of groundwater resources using MODFLOW and DRASTIC models

**DOI:** 10.1038/s41598-023-35496-8

**Published:** 2023-05-22

**Authors:** Saeid Eslamian, Yaghub Harooni, Yaser Sabzevari

**Affiliations:** Department of Water Science and Engineering, College of Agriculture, Isfahan University of Technology, 8415683111 Isfahan, Iran

**Keywords:** Environmental sciences, Hydrology

## Abstract

Groundwater assets are the foremost imperative assets of freshwater accessible to people especially in arid and semi-arid regions. For the investigation of temporal changes in groundwater nitrate pollution and the role of agriculture and other sources in the pollution of groundwater, the information on 42 drinking water wells with suitable distribution in the plain in Bouin-Daran Plain in the center of Iran was used. The results showed that the amount of hydraulic conductivity in the plain for different areas after calibration in steady state was calculated between 0.8 and 34 m/day. After calibrating the model in permanent conditions, the model was calibrated in non-permanent conditions for 2 years. The results showed that in a wide area of the region, the nitrate ion concentration has values of more than 25 mg/L. This shows that the average concentration of this ion in the region is generally high. The highest level of pollution in the aquifer of the plain is related to the southern and southeastern parts of the plain. Due to the agricultural activities with the use of large amounts of fertilizers in this plain, there is a potential for pollution in all of the places, and it requires codified and executive planning for agricultural operations as well as the use of groundwater sources. The DRASTIC vulnerability estimation method is only useful for estimating the areas that have a high potential for contamination and according to the validation tests, it has also provided a suitable estimate.

## Introduction

Groundwater resources are the most important resources of freshwater available to humans. The development of agriculture due to the increase in population and the demand for food security has led to the pollution of groundwater, which is caused by several factors such as soil erosion, and extreme use of fertilizers and pesticides. Failure to apply appropriate management solutions causes complex and bigger problems such as the spread of diseases caused by polluted water, the death of aquatic animals, and the destruction of wetlands and rivers^1,2^.

On the other hand, agriculture is always considered the biggest consumer of freshwater. Water used in agriculture always returns to the surface and groundwater in a chain. However, agriculture is both the cause and the victim of water pollution^3,4^. Therefore, it is important to manage both the development of the agricultural sector and the quality of groundwater resources.

Groundwater refers to the water that is under the surface of the earth and can be collected in wells, tunnels, and drainage galleries. Also, groundwater can flow naturally to the surface of the earth in the form of springs^5^. Groundwater resources are not uniformly distributed in all parts of the earth's solid crust. This issue complicates the process of managing and simulating these resources. The existing equations in the modeling process of the groundwater table can be solved in the form of a mathematical model in two analytical and numerical ways. The complexity of the aquifer system, the heterogeneity of the geological formations, different amounts of pumping and feeding at different times, etc., make the numerical models a replacement for the analytical models in the natural environment^6,7^.

Numerical methods based on computer use become the main tool in solving problems in the natural environment. A series of simplifications should be involved in solving the governing equations of the groundwater flow analytically. In these simplifications, assumptions such as homogeneity and one-dimensional or two-dimensional flow are considered. Analytical methods are usually used in the hydraulics of wells. Numerical solutions are very broad and having access to today's computers, they are more useful than complex analytical solutions^8^. Five numerical methods are used in groundwater modeling, which are: finite difference methods, finite element methods, boundary integral equation methods, comprehensive finite difference methods, and analytical element methods.

Modeling groundwater using mathematical and conceptual models and using software for simulating aquifers and groundwater resource systems. Among the models that are more common in the simulation of aquifer layers, it can be mentioned the different versions of MODFLOW, PMWIN, GWM, GMS, and Visual MODFLOW models, which are created using the finite difference method (FDM)^9^. The finite difference method is used to model the flow and ground aquifer layers. These models have been effectively tested in the groundwater in porous environments and conventional conditions^4^. GMS (Groundwater Modeling System) model is the newest and most comprehensive groundwater modeling software available. Modeling in GMS with two methods of the finite difference and finite elements in two-dimensional and three-dimensional environments and using tools (MODFLOW, MODPATH, MT3DMS/RT3D, SEAM3D, PEST, SEEP2D, etc.) is done^4^.

Nitrate is the most important polluting factor of groundwater, most of which is from agriculture^10^. Groundwater pollution is caused by the addition of nitrate forms to the soil, or through the biological processes, from the conversion of other forms of nitrogen. According to the United States Environmental Protection Agency (USEPA), the maximum permissible concentration of nitrate nitrogen (NO_3_–N) for drinking water is 10 mg/L, which is approximately equivalent to 45 mg of nitrate (N_2_O). This limit according to the World Health Organization and the Union Europe (EU), 50 mg/L of nitrate has been determined. In Iran, 50 mg/L of nitrate has been determined as the maximum allowed concentration in drinking water^11,12^. Therefore, conducting various research in the field of pollution and nitrate concentration prediction is very essential.

From the research done in this field, it can be referred to the study of Pacheco *et al.* In this research, they revised the DRASTIC model to assess the risk of groundwater pollution in the rural mountain watersheds in northern Portugal. Based on the results, the risk of groundwater pollution by nitrate was generally classified as moderate. High-risk areas were areas that are used for agriculture and livestock production^13^. El Baba *et al.* assessed and mapped the groundwater vulnerability and nitrate pollution using the DRASTIC analysis and geostatistics. This research reported a huge difference in the results due to the presence of areas with high and low vulnerability^14^. Moghaddam *et al.* assessed the vulnerability index of groundwater resources using the nitrate concentration prediction approach. The results showed that most of the wells have faced an increase in concentration, which is intensified in the lower areas of agricultural and urban lands, and the vulnerability index showed that the parts of the central aquifer located downstream of agricultural lands and urban areas along the main drain, they are the most vulnerable^15^. Boufekane *et al.* discussed the hybridization of the DRASTIC method to assess the future scenarios of groundwater vulnerability in northeast Algeria. The results of using the DRASTIC method in the reference year of 2010 showed that the high and medium vulnerability classes cover a wide range of the studied area in about 97%. The results for the future groundwater vulnerability prediction showed that groundwater vulnerability changes over time (2010–2030) are closely related to the groundwater depth changes caused by the pumping rates, because the reduction of piezometric level causes the groundwater vulnerability to worsen^16^. Xiao *et al.* discussed Hydrogeochemical insights into the signatures, genesis and, sustainable perspective of nitrate-enriched groundwater in the Piedmont of Hutuo watershed, China. To achieve the sustainable development of groundwater resources in arid and semi-arid foothills, initiatives are recommended, including raising awareness among residents, environmental protection, different water management based on water quality, and targeted water treatment^17^. Xiao *et al.*^18^ Investigated sources, driving forces, and potential health risks of nitrate and fluoride in groundwater of a typical alluvial fan plain. Results indicated that Human inputs/impacts are the dominant forces increasing the nitrate content and salinity of groundwater in the Piedmont region and the residential and industrial lower reaches of the Southeast, posing potential non-carcinogenic risks to various populations through the oral route. Zhi *et al.*^19^ studied the Enrichment mechanism of fluoride and iodine in saline groundwater in the lower flood plain of the Yellow River, northern China. The results of the factor analysis further confirm the opposite behavior patterns of fluoride and iodine in saline groundwater.

In areas with limited surface water resources, the pressure on groundwater is greater, which is due to the gradual decrease in the volume of the groundwater reservoir due to the additional extraction by dug wells and its intensification, especially due to the occurrence of recent droughts on the one hand and pollution caused by the activity. Also, economic issues require accurate knowledge of the aquifer and providing necessary information to exploit groundwater resources. On the other hand, the need to use nitrate fertilizers in agriculture has caused a significant increase in the use of this type of fertilizer. It seems that the use of GMS (MODFLOW) and DRASTIC models in the field of aquifer quality management as a new method can reduce many related problems and costs. Based on the study of different sources, it is possible to understand the importance of investigating nitrate contamination of groundwater sources and the risk of the vulnerability of these sources. The objectives of the present study are: investigating and understanding the temporal changes of nitrate contamination of groundwater in Bouin-Daran plain for one year through sampling once every three months, investigating the role of agriculture and other sources in nitrate contamination of groundwater, the quantitative and qualitative modeling of the plain aquifer using the GMS model, determining the vulnerability potential of the groundwater table in the region using the DRASTIC model and optimizing the weights and scores of the model according to the nitrate concentration of the groundwater, determining the critical points to the vulnerability map tool identified by DRASTIC and providing the suggestions for better management of groundwater in Bouin-Daran aquifer, Iran.

## Materials and methods

### Study area

**Figure 1:**
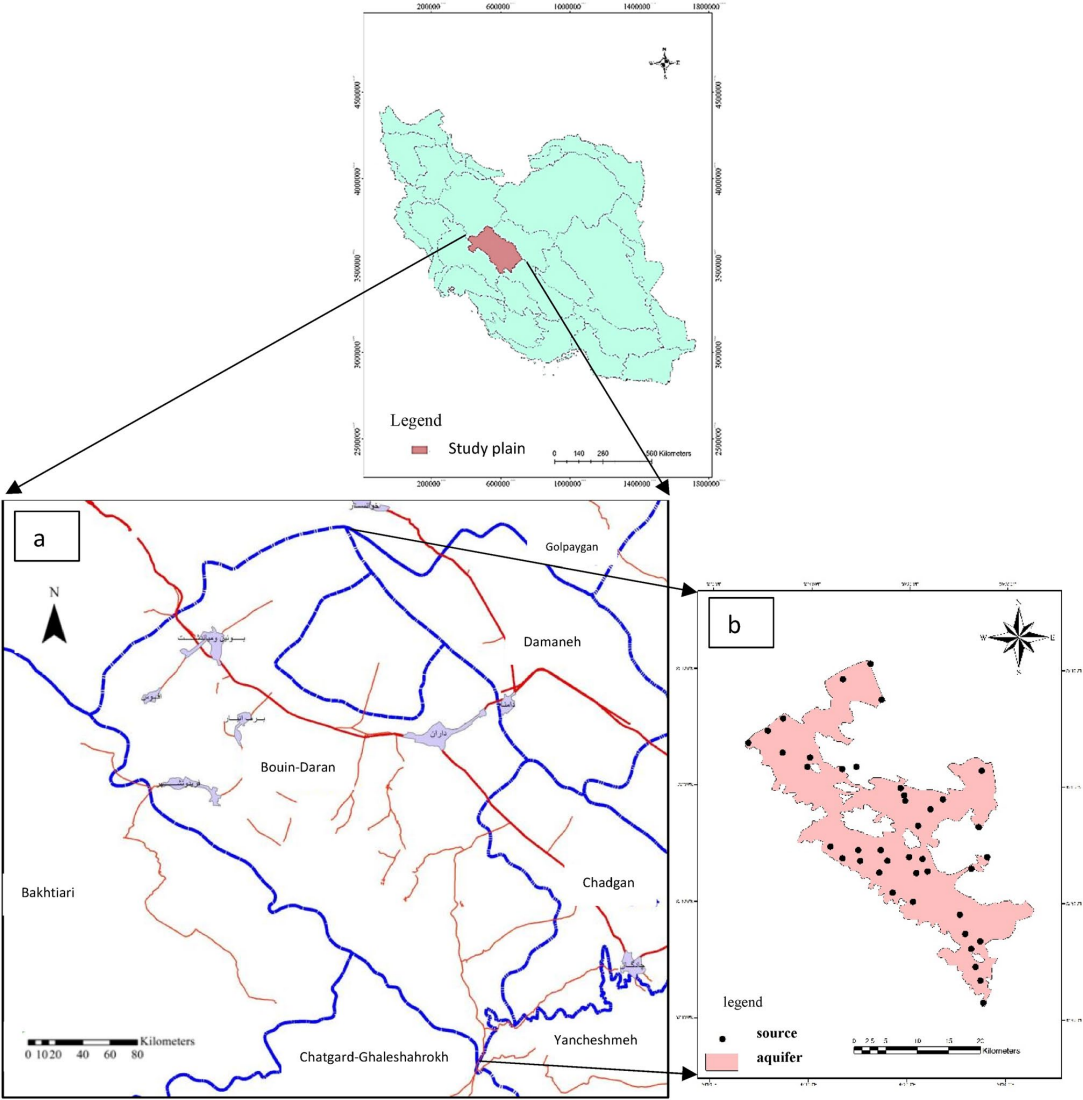
The location of the study area (**a**) and number of samplings of Bouin Daran, Iran using GIS (**b**).

In terms of groundwater investigations, the Gavkhoni Basin has been divided into 21 study areas ^20^, which is the study area of Bouin-Daran in the headwaters area of the basin and the geographical coordinate of east longitude of 50° 8′ to 50° 32′ north latitude of 32° 42′ to 33° 12′. The total size of the study area is about 1262 km^2^, of which 530 km^2^ are highlands and 732 km^2^ are plains. About 660.5 km wide, the plain is covered by an alluvial aquifer. The highest point of this study area is 3735 m in the northern heights and the lowest is 2075 m above sea level in the south of the plain. Figure 1a shows the location of the study area and Fig. 1b shows 42 sampling points of Bouin-Daran. The alluvium containing the groundwater table in this area has a different composition of clay, sand, and gravel and different percentages of these particles can be seen according to the sedimentation conditions at the different depths. Another noteworthy point is the depth and rock type of alluvium in the area, which, based on the drilling logs and observation and exploration wells of the area, is made of marl and schist. Another point is the type of groundwater table. In the study area, there is no impervious layer between the groundwater and the ground surface, and the groundwater table in this area is open. In the northwestern and eastern parts of the study area, the depth of the groundwater level is low and between 10 and 40 m, while in the south, east and southeast, the depth of the groundwater level has increased. The general direction of the groundwater of the region is from the northwest and northeast to the south and east of the region. The most important formation in the hydrogeology of the studied area is the limestone formations of the Cretaceous period. In the study area of Bouin-Daran, three selected aqueducts, three selected spring mouths, and eight selected wells have been selected by Isfahan Regional Water Company as the selected water sources, and selected springs and aqueducts are generally located in the valleys and on the edge of the highlands and selected wells are located in the plain. It should be mentioned that to better analyze the quality of groundwater in the study area, the results of chemical analysis of three selected wells and a selected aqueduct in the study area of the domain have also been used. Due to the small number of the selected water sources and their inappropriate distribution in the plain, to investigate the quality of groundwater in the study area, sampling results from several agricultural wells in the area as well as drinking water sources in the villages of the area were also used. Table 1 shows the characteristics and results of the chemical analysis of selected water sources in the region.

**Table 1:** Chemical analysis related to water wells.

Water source	Name	Longitude	Latitude	Year	EC (ds/m)	TDS (meq/l)	pH	HCO_3_^−^ (meq/l)	Cl^−^ (meq/l)	SO_4_^2−^	Ca	Na	K
Fountain	Freydoun Shahr	416700	3645300	1384	210	137	8.10	134.22	3.55	0.48	36.07	2.30	0.39
deep well	Afus	417335	3656347	1390	355	231	7.70	201.33	14.18	5.28	50.10	2.30	0.04
Aqueduct	Morgh deraze	420769	3652273	1386	188	122	8.60	103.72	3.55	0.48	28.06	2.30	0.39
deep well	Mirabad	425933	3661798	1390	399	259	8.00	213.54	17.73	5.28	36.07	2.30	0.04
Aqueduct	Shah Enayat Sanghrah	427347	3665693	1391	341	222	8.20	201.33	14.18	0.48	56.11	2.30	0.04
Deep well	Dehsour	430038	3639649	1390	482	313	8.10	207.43	24.82	53.31	42.08	2.30	0.04
Deep well	Ghalee Malek	436303	3636631	1390	443	298	7.80	231.84	14.18	67.72	72.14	2.30	0.04
Deep well	Khoygan Sofla	439516	3631271	1390	435	281	7.30	219.64	14.18	10.09	50.10	2.30	0.39
Deep well	Nama Gard	440735	3649510	1390	369	240	8.10	189.13	21.28	10.09	48.10	2.30	0.04
Aqueduct	Belmir	446028	3637508	1390	307	200	8.20	189.13	7.09	0.48	32.06	2.30	0.04
Deep well	Eskandari	448075	3631424	1390	381	248	8.10	189.13	17.73	14.89	58.12	2.30	0.04
Fountain	Daran	448297	3649606	1390	237	154	7.40	122.02	14.18	5.28	42.08	2.30	0.04
Fountain	Geshnizgan	449058	3626914	1391	588	382	7.70	292.85	14.18	29.30	72.14	11.50	0.04
Deep well	Dareh bid	452595	3656461	1390	452	294	7.90	189.13	10.64	38.90	52.10	6.90	0.04

The reason for choosing these ions is that due to the heavy agriculture and the nature of the fertilizers used, as well as the special condition of the bedrock and groundwater in this plain, it seems that the most important chemical pollutant of the groundwater is nitrate^21^. However, there is a non-point due to the use of organic and mineral fertilizers in this area. On the other hand, the supply of drinking water to the cities and villages of the plains from groundwater made the management of groundwater be considered for the changes of groundwater in this region. to measure the nitrate in the plain and considering the selection conditions of the sampling wells, 42 drinking water wells were selected with the proper distribution in the plain, and chemical analyzes were performed on the samples taken in three-month intervals. After selecting the sampling wells in the different study areas to distribute the nitrate pollution in groundwater, sampling of these wells started in September 2013. Also, to check the trend of nitrate pollution changes, sampling was continued at 3-month intervals in four stages until September 2014. At each stage, sampling of water from the wells was done and the samples were transported to the laboratory in 1-L plastic containers and kept in the refrigerator until the time of chemical measurement to prevent biological activities and changes in the chemical properties. After being transferred to the laboratory, the well-water samples were immediately chemically analyzed according to the standard laboratory methods. Nitrate ion was measured by Southern ion selector electrode model 3310. The potential difference between the two sides of the nitrate electrode membrane is measured compared to the potential difference of a reference electrode. The device measures the activity of nitrate ions. This electrode is capable of measuring nitrate in the range of 7*10^(−6)^ up to 1 molar. It should be noted that this is if there are no interfering ions in the solution. The most important disturbing ions in the nitrate electrodes solution are chlorine, bicarbonate, acetate, sulfate, and fluoride, while bicarbonate and chlorine ions cause more disturbance. Despite the problems related to the interference of ions, the method of nitrate measurement with the help of an electrode is superior to the other methods due to the associated higher accuracy and saving both time and cost. After the desired measurements, the relevant calculations are done.

### Construction and calibration of the MT3D Code

#### Making a mathematical model (determining the boundary of the aquifer)

**Figure 2:**
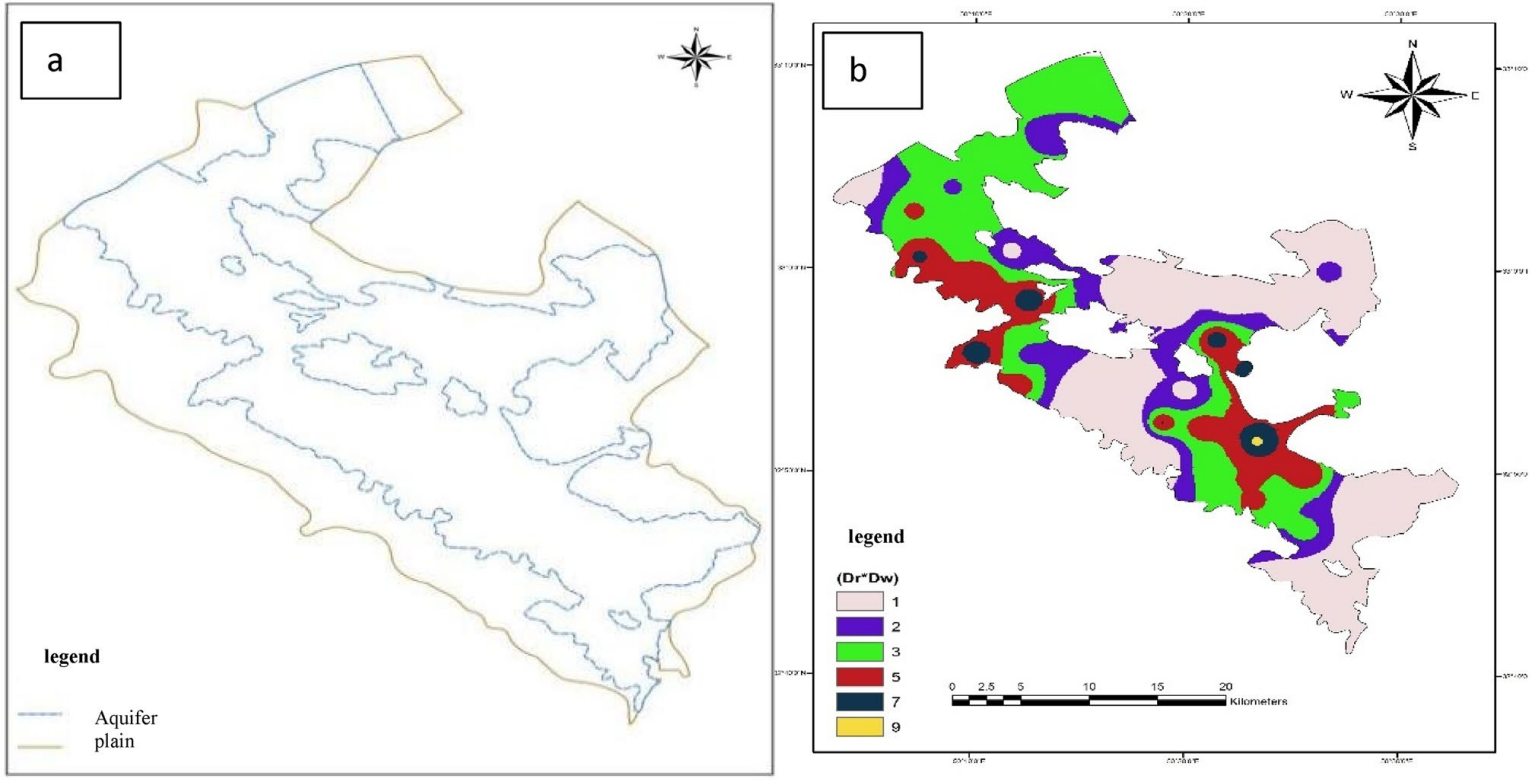
Map of Bouin-Daran Plain aquifer area (**a**) and Depth of water table using GIS (**b**).

The area of the aquifer of the plain was determined by examining the logs of wells in the area, geological maps, and the DEM map of the area, and the area of the aquifer and the Bouin-Daran plain is shown in Fig. 2a. After determining the boundary of the aquifer, the conceptual model of the aquifer was prepared according to the available information. This stage includes determining the boundary of the aquifer, determining the inflows to the aquifer, determining the outflows from the aquifer, the number of stratigraphic layers of the aquifer, creating the aquifer feeding layer, creating the hydraulic conduction layer of the aquifer, creating the layer of observation wells of the aquifer, creating the special irrigation layer of the aquifer, creating the layer of pumping wells of the aquifer. Creating the topographic layer of the aquifer surface makes the topographic layer of the aquifer floor. Then, the 3D network of the MODFLOW model was created and the information from the conceptual model was converted into a 3D mathematical model^22^.

#### Model calibration in steady state

To calibrate the model in a steady state, the piezometric data of March 2003 were used. In this case, the values of the hydraulic conductivity and feeding were calibrated by determining and changing the different areas manually and using the PEST code for the aquifer. The accuracy of the calibration process was determined by comparing the calculated values and the observed values of the water level in the piezometers. For this purpose, two indices of the coefficient of determination (R) and RMSE error were used^23^.

#### Model calibration in unsteady state

After calibrating the model in permanent conditions, the model was calibrated in temporary conditions for a period of 2 years (April 2003 to March 2004). At this stage, the feeding and special watering values were recalibrated and the hydraulic conductivity values were also modified^24^.

#### Validation of the model

After calibrating the model in temporary conditions, the model was validated in one-year (April 2005 to March 2005).

#### Construction and calibration of the qualitative model of the aquifer in the MT3D code

After creating the quantitative model, the qualitative model of the aquifer was prepared using the MT3D code^25^. For this purpose, nitrate pollutant was selected as the parameter to be evaluated and pollutant transport factors, including transport, diffusion, and spreading, were introduced to the model. Then, the observation wells for nitrate measurement were entered into the model. Finally, the model was calibrated and validated for one year.

### DRASTIC model

#### Drastic model parameters

First, information such as geological maps, meteorological records, piezometer records, exploration wells, and well logs was collected to build the layers of each parameter in the Arc GIS software^26^. These data were collected from different sources and in different formats. The database for point format data was first prepared by the Excel software, then entered into the GIS. The imaging system of all of the information layers is UTM zone 38, and the base level is WGS 1984. Also, spatial analysis functions were performed on the data to convert them into a map, including the topological analysis functions such as clipping, merging, and overlay, and surface analytical functions such as extracting the slope from the digital model named the height^27^. IDW (Inverse Distance Weighted) transformation was used for interpolation. This is because the error of this method based on the RMSE and cross-validation is less than the other methods such as kriging, co-kriging, and spline. Using the Spatial Analyst program, the maps were divided into the desired classes and after preparing the basic maps using the features of this program regarding the raster operation, the final map was calculated according to the DRASTIC index.

#### Data sources used for each model parameter

Groundwater Depth Layer (D).

The depth of the groundwater expresses the depth from the ground surface of the water table level. The depth of the water table, along with the characteristics of the unsaturated zone, affects the movement time of solid or liquid pollutants that are transported by water and the dilution time of pollutants in the unsaturated zone^28^.

Figure 2b shows the obtained layer after valuation. The groundwater depth layer of the region is placed in seven classes^1–3,5,7,9^. This rating was done for each cell with dimensions of 100 m.

#### Net feed layer (R)

Usually, the more nutrition there is, the greater the potential for groundwater pollution. Piscopo’s (2001) method was used to prepare the feeding layer^29^. Piscopo's equation for calculating the feeding potential of an area is as follows:

$${\text{Nutrition score}} = {\text{soil permeability score}} + {\text{rainfall score}} + {\text{slope percentage score}}$$

**Figure 3:**
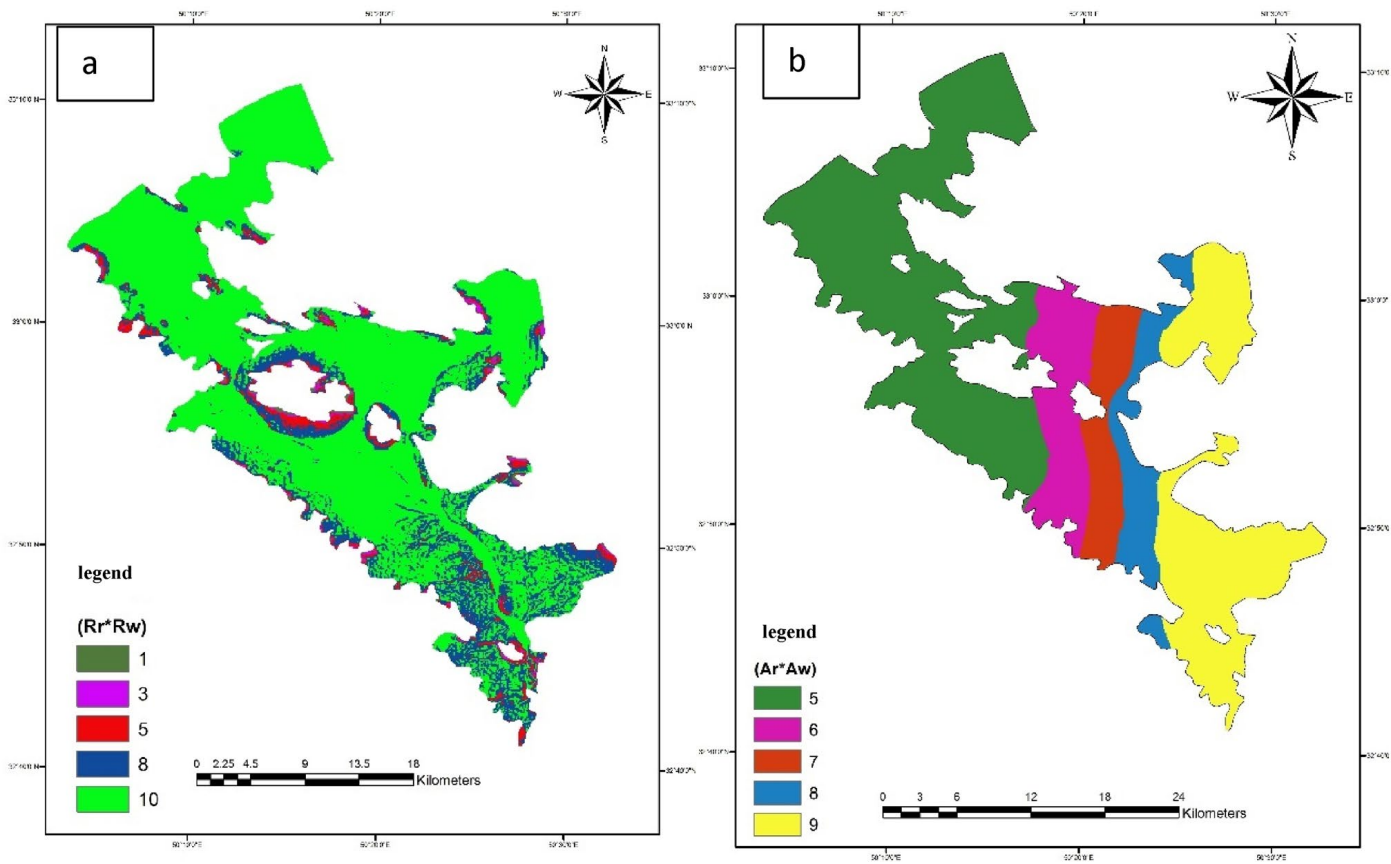
Valued map of net nutrition (**a**) and Valued map of aquifer environment using GIS (**b**).

To calculate the amount of nutrition, a digital elevation model (DEM) of the area was prepared (using DGN maps of the area with a scale of 1:25,000 prepared by the country's mapping organization). Then the slope of the studied area was extracted using the prepared DEM and finally, all of the maps were converted into a raster format. The nutrition map was prepared by overlaying the slope and soil map along with the rainfall score of the area (Fig. 3a).

#### Aquifer environment layer

To prepare the aquifer environment layer, the logs of seven wells in the region, which were prepared by the study office of the regional water company, were used for exploratory and exploitation wells in the region. The polygons related to the aquifer environment were prepared using the Xtools program in the Arc GIS software (Fig. 3b). Then, the map of the aquifer environment was prepared in raster format with a cell size of 100 m so that it can be used in the other stages of calculations.

#### Soil layer (S)

**Figure 4:**
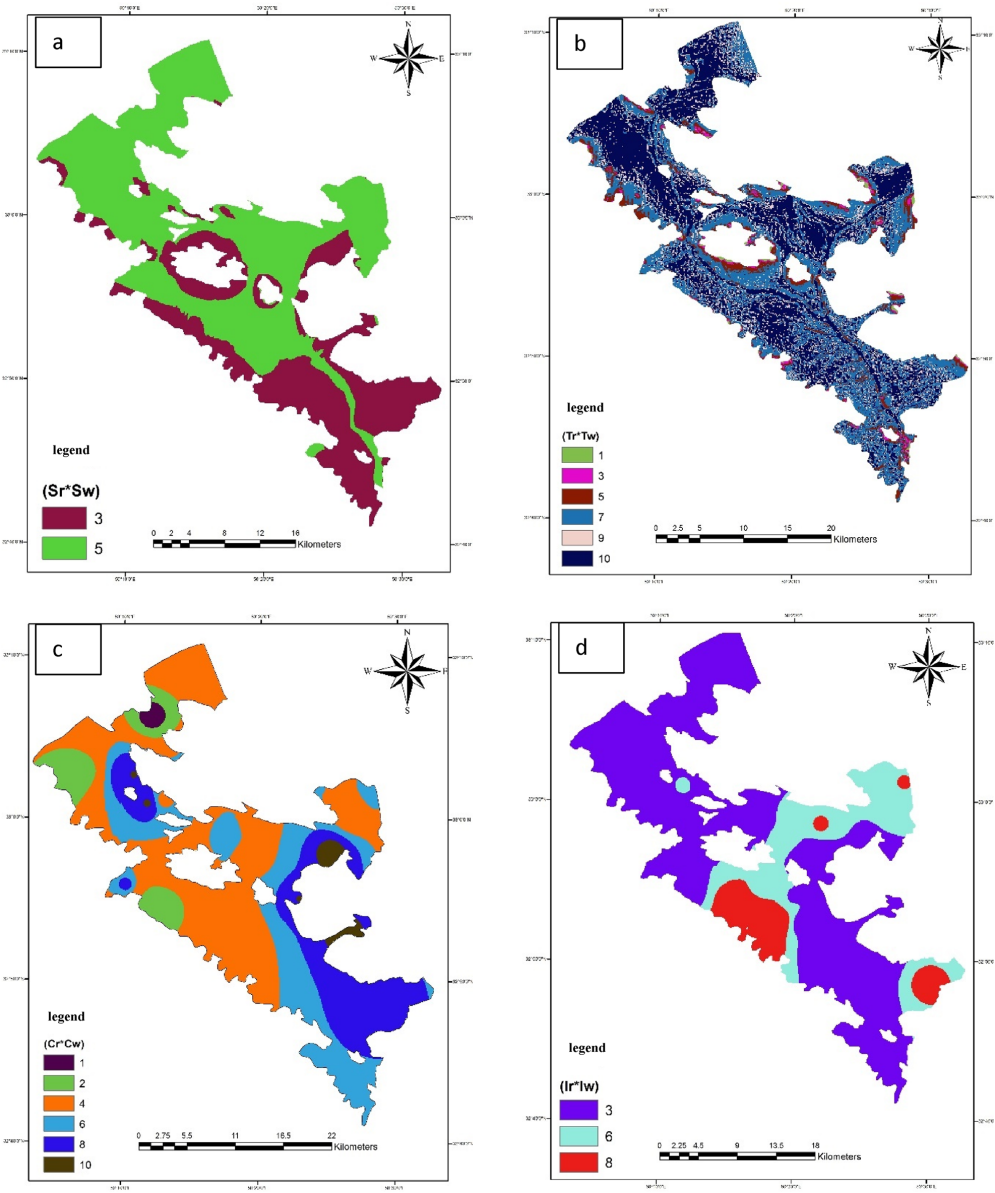

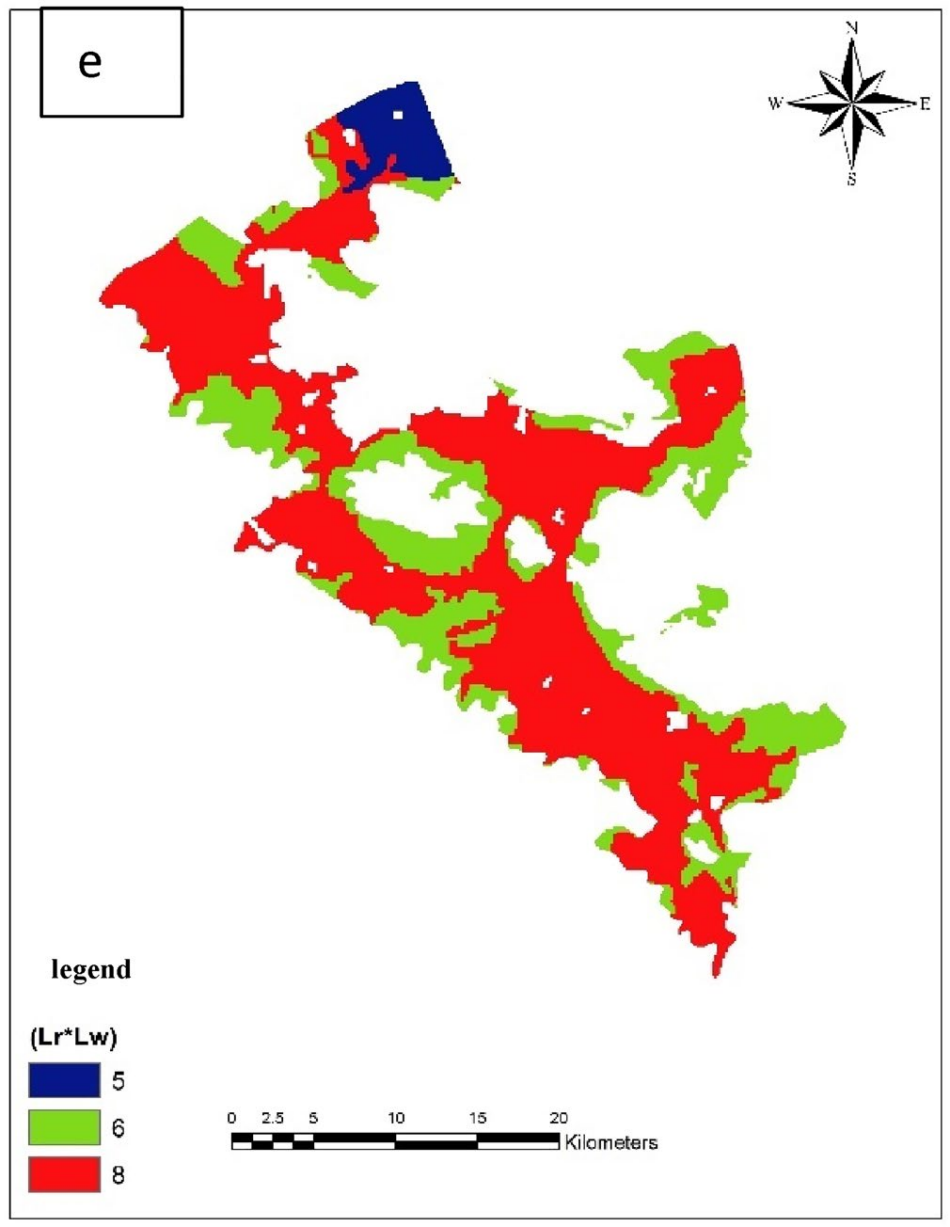
Valued map of soil layer (**a**), Valued topography map (**b**), Valued map of the unsaturated environment (**c**), Valued map of hydraulic conductivity (**d**), and Valued map of land use in Bouin-Daran aquifer using GIS (**e**).

To prepare the soil layer, the map of the region with a scale of 1:50,000 prepared by the Agricultural Research and Natural Resources Organization was used. The soil map was first scanned, then it was referenced and digitized, and the grades of each polygon were applied using the drastic method (Fig. 4a).

#### Slope layer or topography (T)

First, a digital height model of the area was prepared and then the slope map was extracted from this model. Finally, the obtained slope map was graded to prepare the topographic layer (Fig. 4b).

#### Impact layer of the unsaturated zone

The unsaturated environment layer, like the aquifer environment layer, was determined and valued from the logs of piezometers, the logs of exploratory wells in the area, the geographical location of the logs, and the type of the unsaturated zone. The polygons related to the unsaturated zone map were prepared using the Xtools program in the Arc GIS software. Then, the map of the unsaturated zone was prepared in a raster format with a cell size of 100 m so that it can be used in the calculation steps like the other layers (Fig. 4c).

#### Hydraulic conduction layer (C)

Information and geographical points related to the transferability coefficient and alluvial thickness of the plain were prepared from existing maps. Then, by subtracting the alluvial thickness map and the water surface depth map, the saturated thickness map was obtained by using the Raster calculator function, and after dividing the saturated thickness map of the aquifer, the hydraulic conductivity map of the aquifer was obtained^30^. The resulting map was graded according to the drastic index (Fig. 4d).

#### Land use map

Taking into account the watershed land use map in Daran Plain, the following map was obtained. As can be seen, the largest area of the plain is related to the water lands where potatoes are cultivated (Fig. 4e).

#### Data collection related to calibration and validation of DRASTIC model

To calibrate the model. DRASTIC was used from data collected from 44 wells that are evenly distributed on the surface of the aquifer and the plain.

## Results and discussion

**Figure 5:**
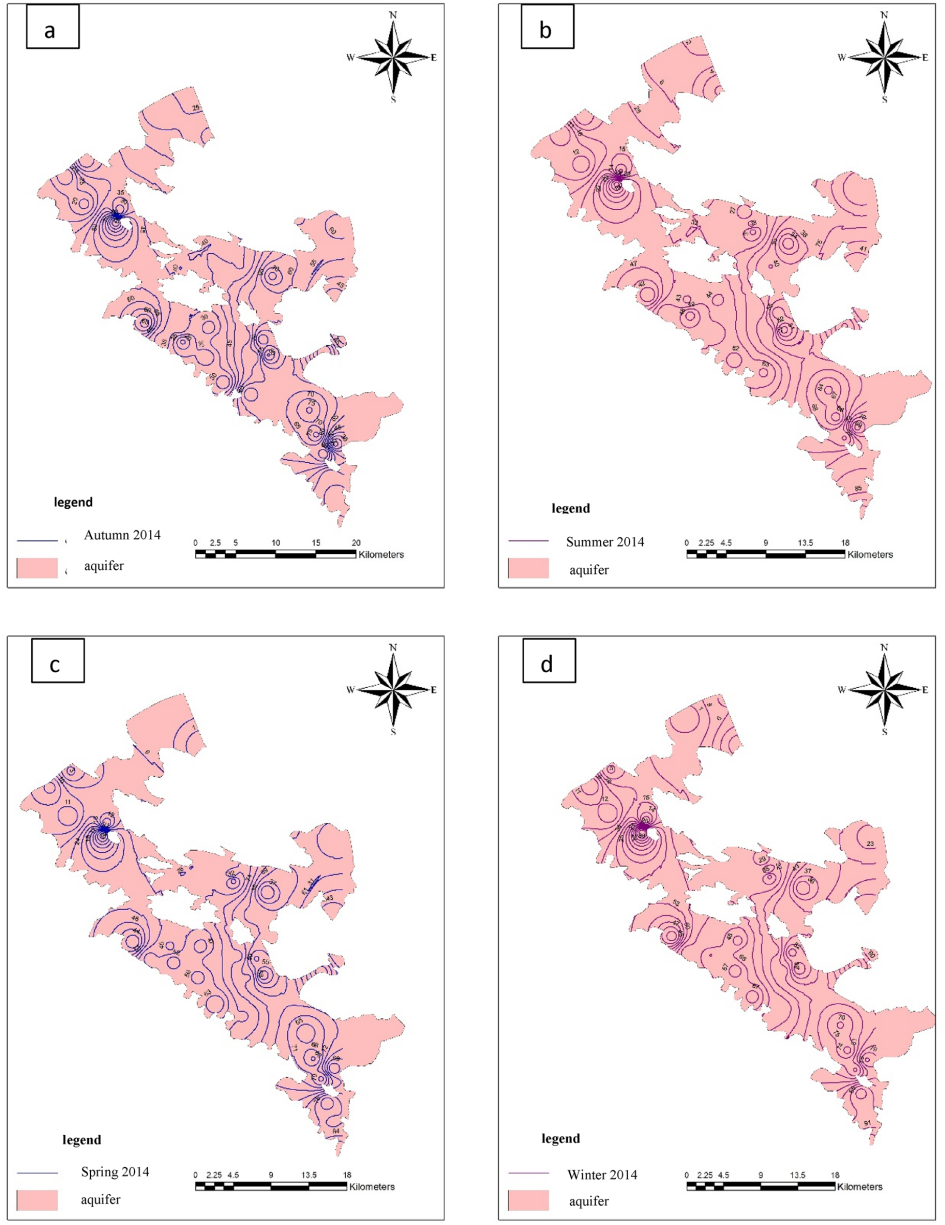
Hemi nitrate lines Autumn 2014 (**a**), Summer 2014 (**b**), Spring 2014 (**c**), and Winter 2014 using GIS (**d**).

The maps in Fig. 5 show the parallel curves of nitrate in the study area. Based on that, nitrate ion concentration shows high values in different seasons in the eastern and southern half of the region. According to the maps in Fig. 5, in a wide area of the region, the nitrate ion concentration has values of more than 25 mg/L. This shows that the average concentration of this ion in the region is generally high. Considering that, this ion generally has a low concentration in groundwater, the existence of high concentrations in the groundwater of any region can be due to various reasons, in such cases this ion is known as an element that pollutes the groundwater. For this reason, the distribution and possible reasons for the increase in the concentration of this ion in the region will be studied and investigated as the pollution of the groundwater table in the region.

### The results of the MODFLOW model

#### Mathematical model in MODFLOW

**Figure 6:**
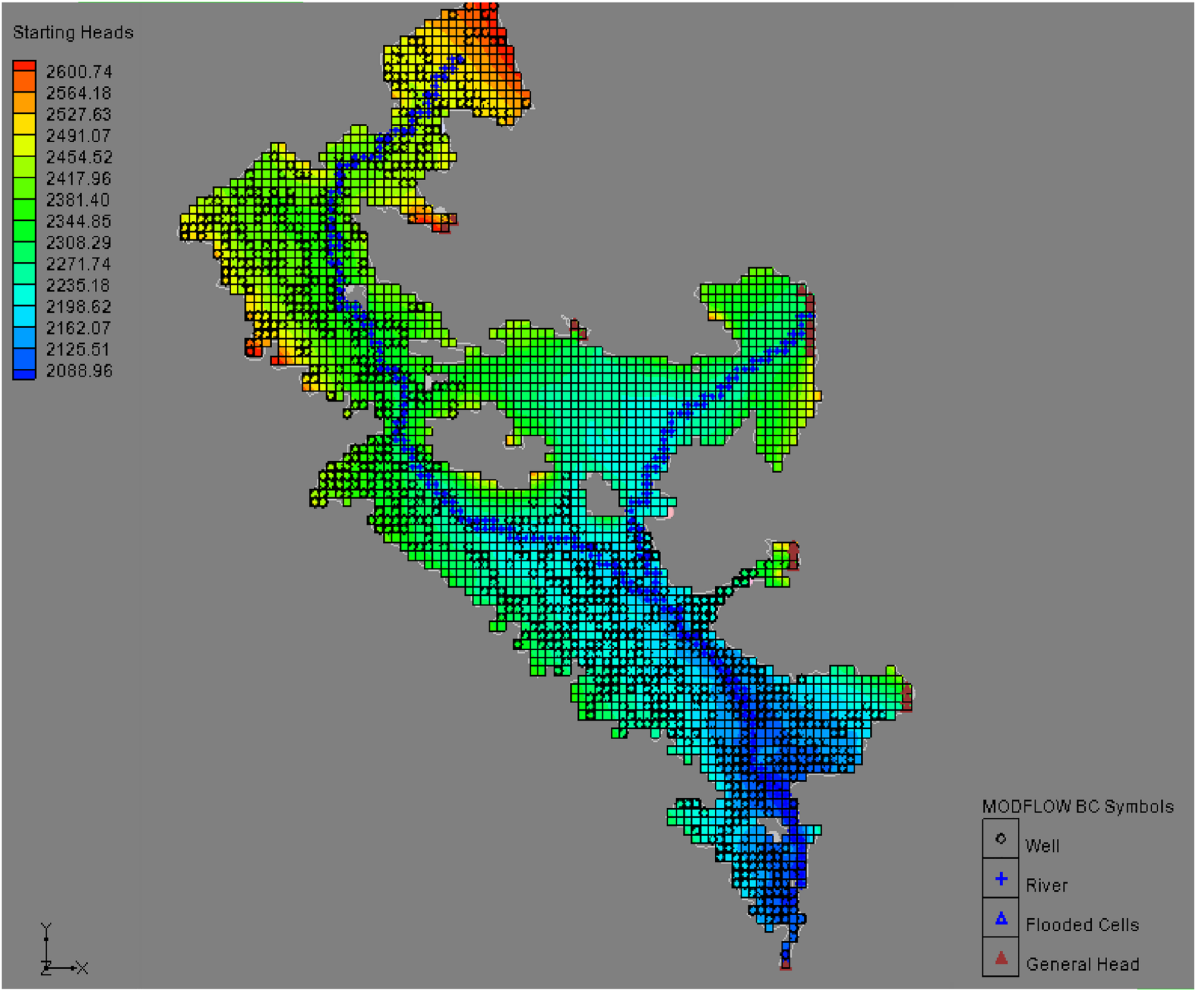
Map of created network and main components of Bouin-Daran aquifer using MODFLOW.

The 3D network of the MODFLOW model was created and the information from the conceptual model was converted into a 3D mathematical model. The created three-dimensional model consists of 12,000 computational cells (with dimensions of 500*500 m), which according to the shape of the aquifer, 2688 cells are the active cells and the rest are inactive cells. Figure 6 shows the 3D model created in MODFLOW.

#### Model calibration in steady state

**Figure 7:**
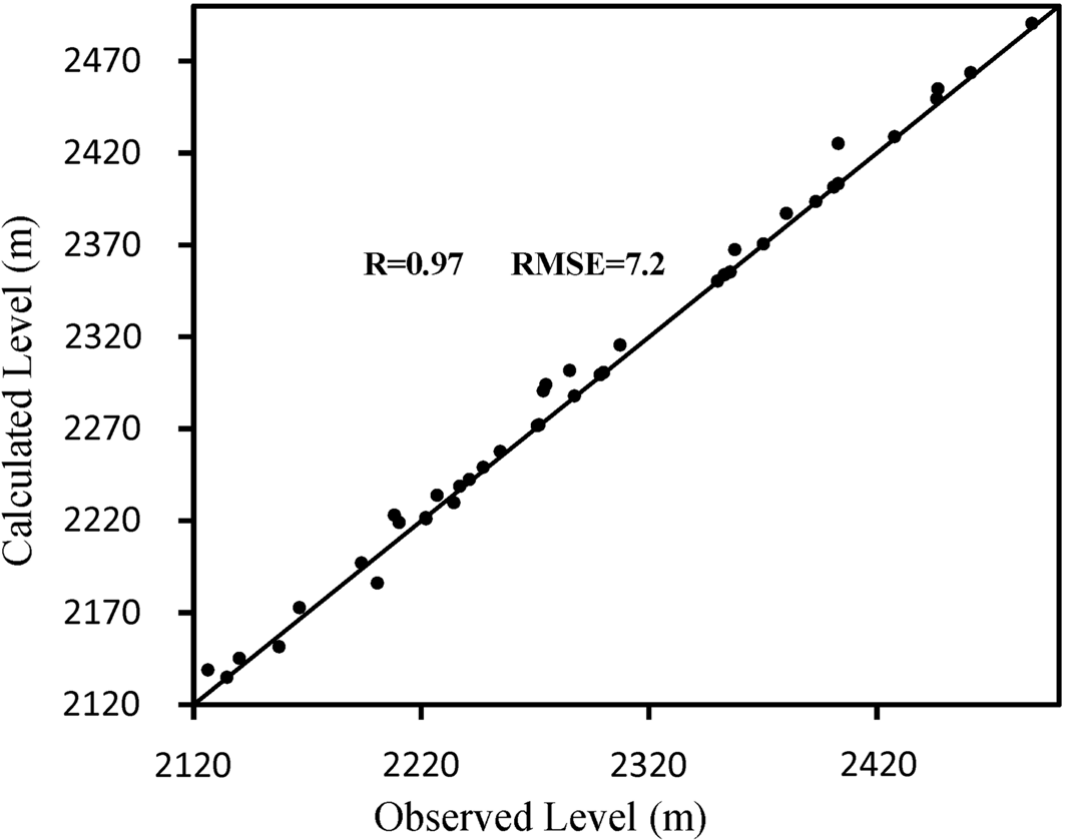
Calculated and observed values of the water level in steady state.

**Figure 8:**
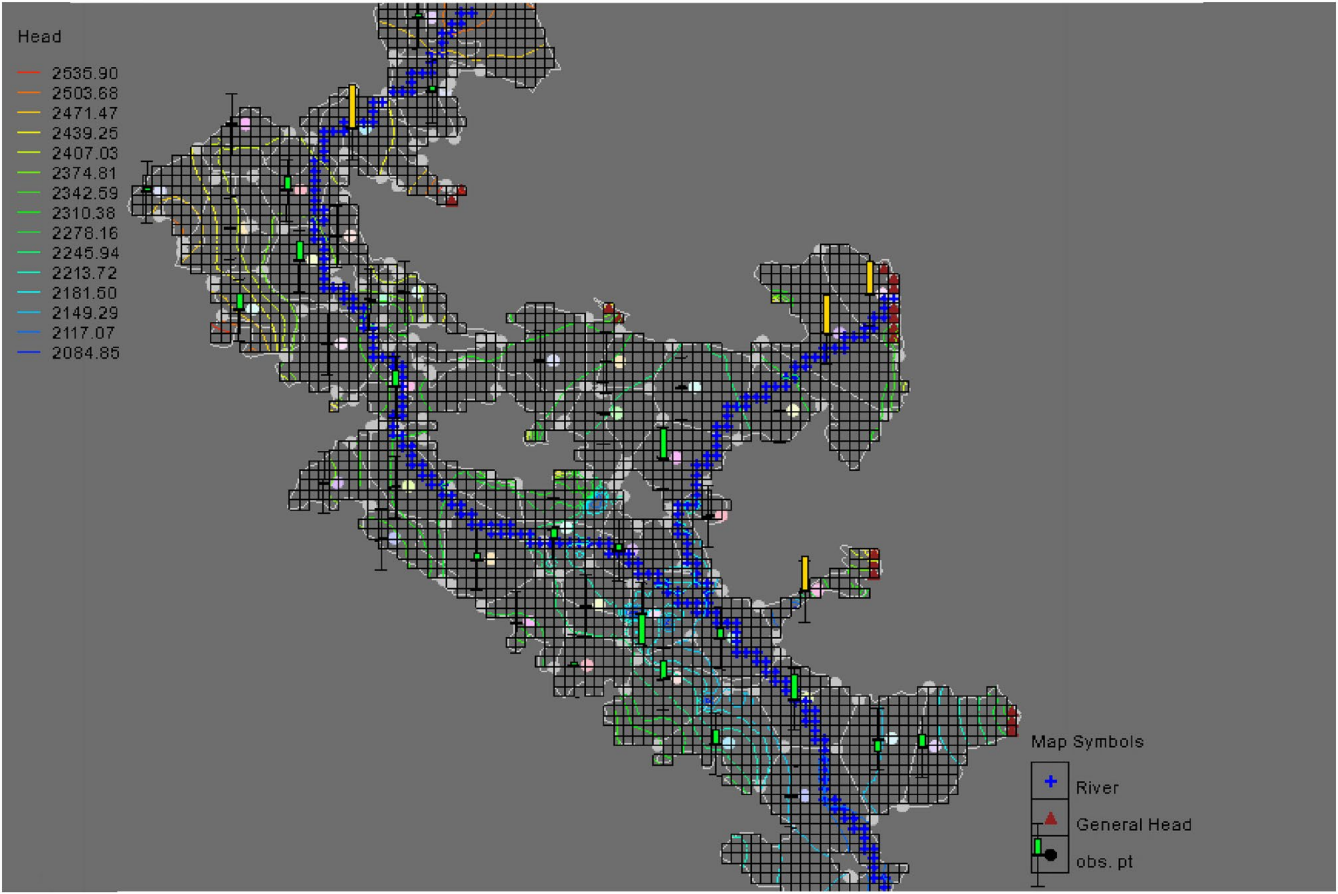
Calibrated model map of Bouin-Daran aquifer using MODFLOW.

The amount of hydraulic conductivity in the plain for different areas after calibration was calculated between 0.8 and 34 m per day. The accuracy of the calibration process was determined by comparing the calculated values and the observed values of the water level in the piezometers. For this purpose, two indices of coefficient of determination (R^2^) and RMSE error were used. The graph comparing the observed and final calculated values in the steady state calibration is shown in Fig. 7. Figure 8 shows the map of the calibrated model of the aquifer along with the piezometers in the area. In this figure, the accuracy of the estimation of the water level in piezometers is indicated in three colors: red, yellow, and green. The piezometers shown in green are calibrated within the ideal range in terms of error variance. Also, yellow and red piezometers indicate moderate and poor calibration, respectively.

#### Hydraulic steering in steady state

**Figure 9:**
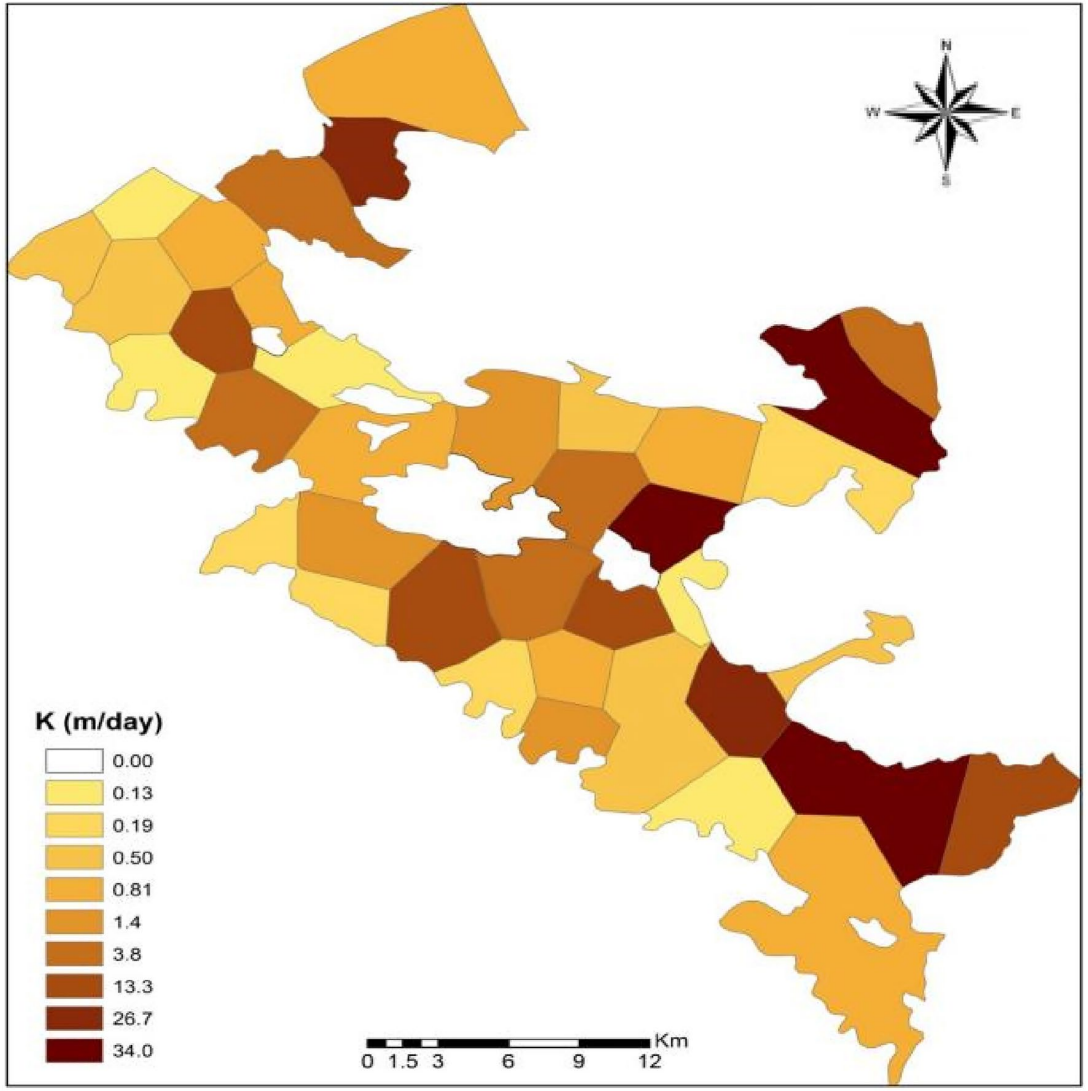
Scatter map of hydraulic conductivity values of Bouin Daran aquifer, Iran using GIS.

Figure 9 shows the map of hydraulic conductivity values obtained from model calibration. As can be seen, the final values of hydraulic conductivity in the aquifer vary between 0.2 and 34 m per day.

#### Model calibration in non-steady state

**Figure 10:**
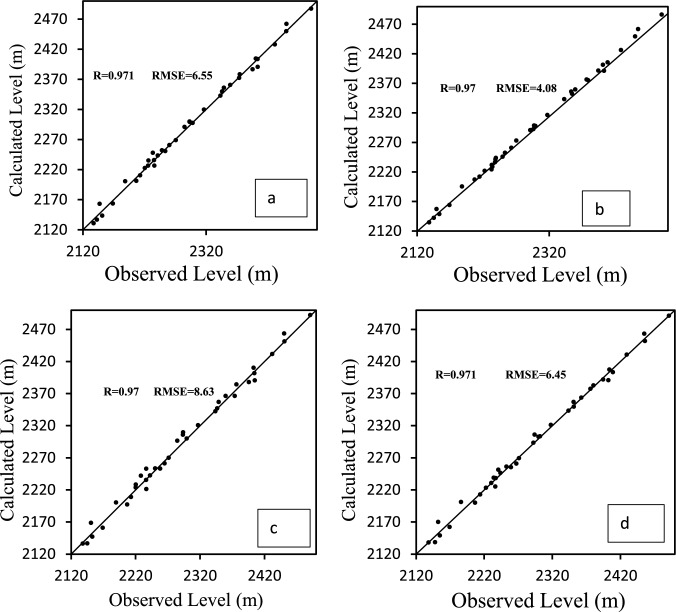
Calculated and observed values of the stability level in a non-permanent state (September 2003) (**a**), (April 2003) (**b**), (September 2004) (**c**) and (April 2004) (**d**).

After calibrating the model in permanent conditions, the model was calibrated in non-permanent conditions for a period of 2 years (April 2003 to March 2004). At this stage, the feeding and special watering values were recalibrated and the hydraulic conductivity values were also modified. The results of comparing the calculated and observed values in representative months are shown in the graphs of Fig. 10. As can be seen, the model has been well recalibrated and the model has estimated the water level values with acceptable accuracy.

#### Mathematical model validation

**Figure 11:**
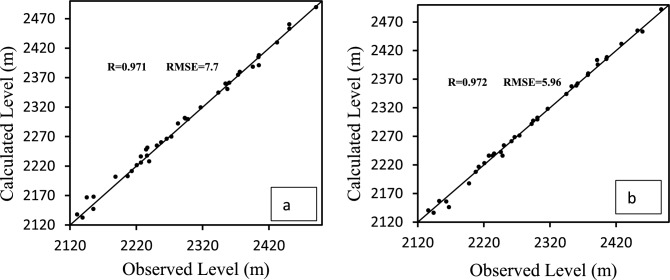
Calculated and observed values of water level in the validation period (September 2005) (**a**) and (April 2005) (**b**).

After calibrating the model in a non-permanent condition, the model was validated in one year (April 2005 to March 2005). The results of comparing the calculated and observed values for April and September are shown in the graphs of Fig. 11a,b. As can be seen, the model has well estimated the values of the water level in the calibration stage. Therefore, the calibrated model can be used for different purposes with confidence.

#### Construction and calibration of the qualitative model of the aquifer in the MT3D code

**Figure 12:**
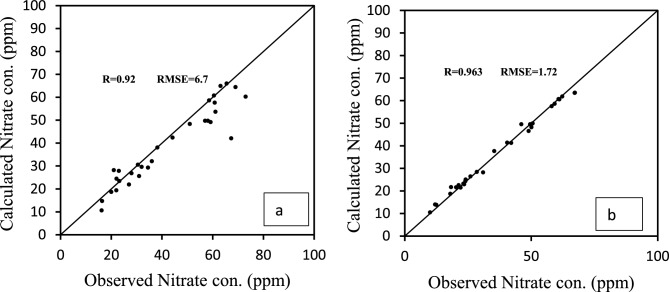
Calculated and observed values of nitrate concentration in the qualitative model (May 2012015) (**a**) and (August 2014) (**b**).

The results showed that due to the vastness of the studied area as well as the high volume of groundwater input and output in horizontal and vertical sections, nitrate changes due to the spreading process are insignificant. In general, the results showed that the qualitative model of the aquifer has very low sensitivity to the changes in the distribution coefficient, diffusion coefficient, and absorption isotherms, and the nitrate changes in the prepared model are the most sensitive to the changes in the nutritional nitrate concentration. The graph comparing the calculated and observed nitrate values in August 2014 and May 2015 is shown in the graphs of Fig. 12a,b. As can be seen, the calibrated model predicts the nitrate concentration values with appropriate accuracy.

**Figure 13:**
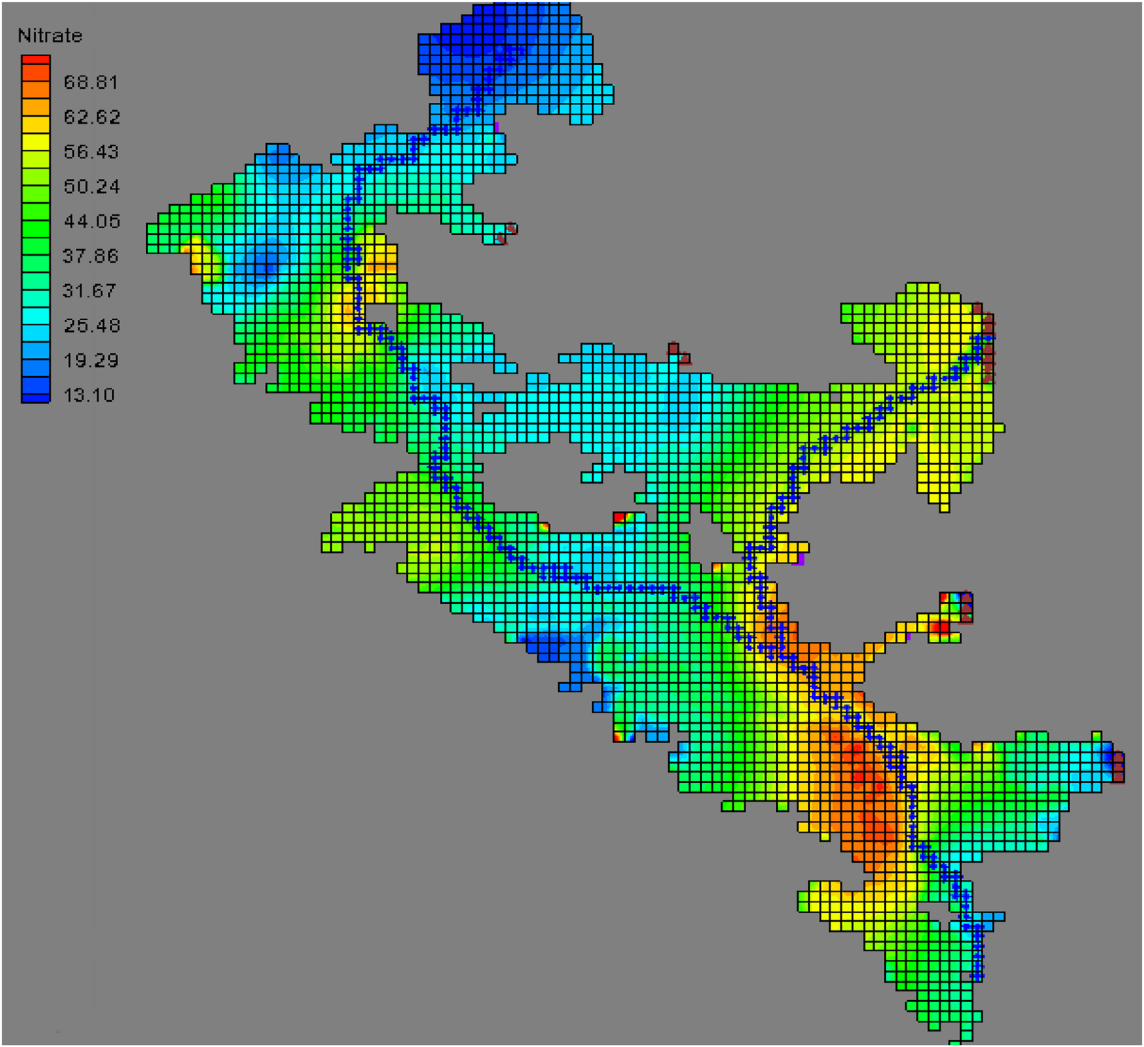
The distribution map of nitrate sediments on the surface of the aquifer (July 2015) using MODFLOW.

Figure 13 shows the distribution of the nitrate concentration obtained from the model in different areas of the aquifer in July 2015. As can be seen, the southern areas of the plain, which are marked with red color, have a high level of nitrate concentration, and this area and the other areas with red and yellow colors are among the areas with high vulnerability and risk.

### DRASTIC model

#### Inherent vulnerability

**Figure 14:**
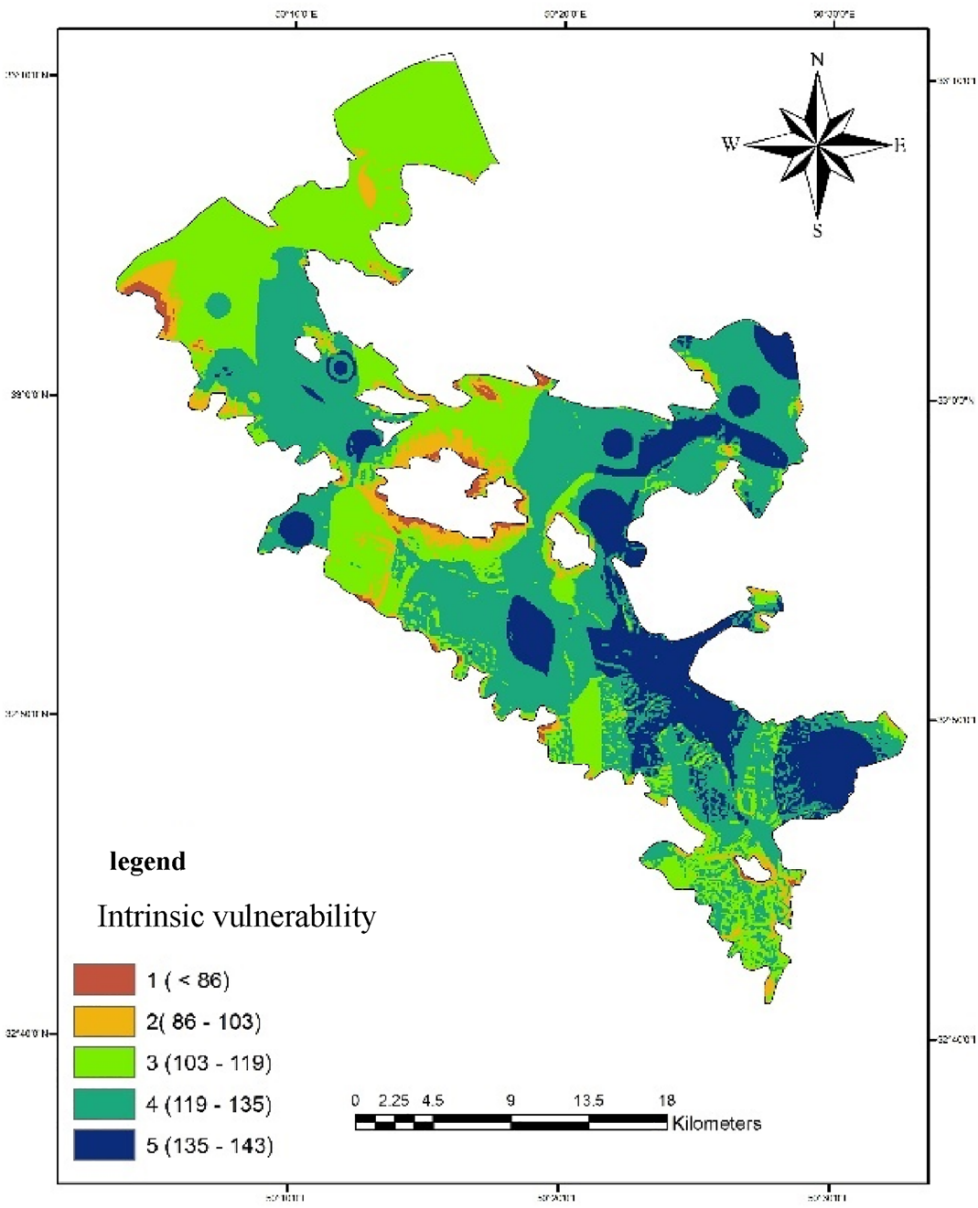
Intrinsic vulnerability map of the aquifer in Bouin-Daran plain using GIS.

In determining inherent vulnerability, groundwater vulnerability is evaluated regardless of surface contamination. In other words, the vulnerability is independent of the user and any occurrence of the pollutant. As it is clear from Fig. 14, areas without risk of pollution and areas with low pollution are located on the slopes and the edge of the heights. Also, the areas located in the northern and central parts of the plain have moderate vulnerability. A very important point is that the eastern and southeastern areas of the aquifer are exposed to a lot of pollution due to the shallow depth to the water level, as well as the closeness of the bedrock to the ground surface and good hydraulic conductivity.

#### Special vulnerability

**Figure 15:**
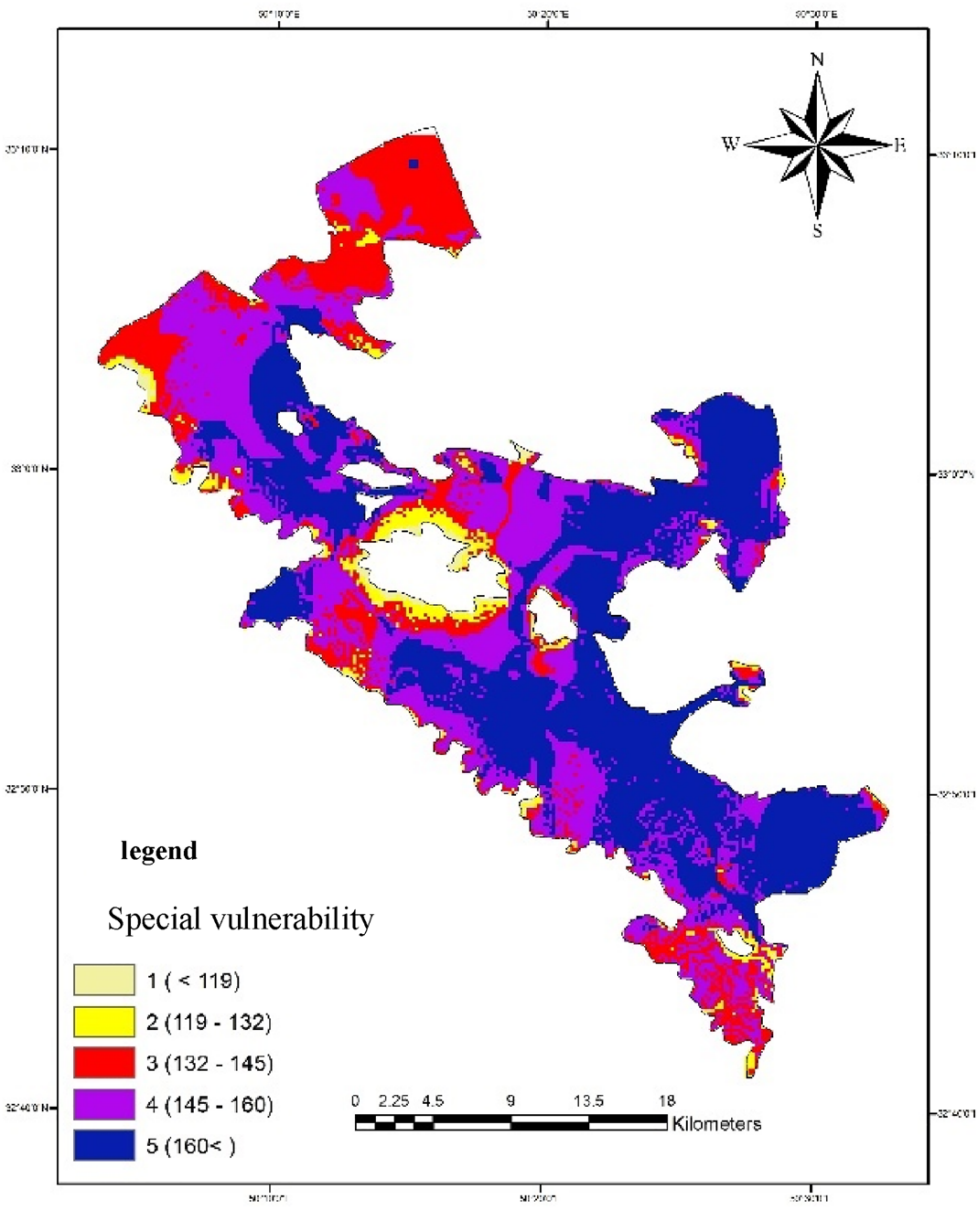
Special vulnerability map of the in Bouin-Daran aquifer, Iran using GIS.

By adding the land use parameter (L) to the inherent vulnerability parameters, the special vulnerability index is obtained. According to Fig. 15 and taking into account land use and taking into account that in aquifer crop areas, potato is the dominant crop, the area that is exposed to high and very high pollution, the east, southeast, and parts of the center of the plain. Another noteworthy point is that, in general, it can be said that the Bouin-Daran plain aquifer has medium to very high vulnerability potential.

## Conclusion

The present study was carried out to provide a model for qualitative investigation and qualitative control of the nitrate situation in the Bupin-Daran Plain aquifer, as well as identifying the points susceptible to pollution such as nitrate. The main findings are as follows:

1. The highest amount of pollution in the aquifer of the plain was related to the south and southeast parts of the plain.
2. The north and northeast areas of the plain had medium to low pollution potential and had not been affected by nitrate pollution at the moment.
3. Due to the agricultural activities with the use of large amounts of fertilizers in this plain, there was a potential for pollution in all of the places, and it requires codified and executive planning for agricultural operations as well as the use of groundwater sources. It is required as drinking water.
4. The DRASTIC vulnerability estimation method was only useful for estimating the areas that have a high potential for contamination and according to the validation test. The ability of the models presented in this research to determine the areas of the wells that are more vulnerable to pollution is one of the problems of the traditional methods of aquifer vulnerability assessment, which is the inability to predict the effect of the spread of pollution in the vulnerable areas of the aquifer on the pollution of valuable water sources such as Drinking water wells need to be fixed. The identification of these areas is very important for the design of the groundwater quality monitoring network, as well as land use studies and land use determination.

## Data Availability

If someone wants to request the data should be contacted by the corresponding author.
